# Calciphylaxis: A Mimic of Vasculitis

**DOI:** 10.31138/mjr.130424.amo

**Published:** 2025-01-21

**Authors:** Dilara Bulut Gökten, Rıdvan Mercan

**Affiliations:** Department of Rheumatology, Tekirdag Namik Kemal University, Turkey

**Keywords:** calciphylaxis, calcification, arterioles, end stage renal disease

## Abstract

Calciphylaxis, which literally means ‘protection through calcification’, is a fatal disease characterised by ischemic necrosis of cutaneous tissue resulting from vascular intimal fibroplasia, thrombi, and calcifications in the arterioles. The pathophysiology involves the accumulation of calcium in the skin, vascular space, and adipose tissue due to abnormal serum levels of calcium, phosphorus, and parathyroid hormone (PTH), particularly in patients with end-stage renal disease (ESRD). The clinical presentation typically involves severe ischemic and neuropathic pain, which may occur independently of skin lesions. There are no specific diagnostic criteria or laboratory tests; the disease is primarily recognised based on clinical findings. There is no definitive information on treatment due to the incomplete understanding of its mechanism; therefore, the prevention of calciphylaxis is of paramount importance. Upon examination of cases in the literature, a mortality rate of 34% is observed despite appropriate treatment and care. Given its rarity, calciphylaxis is prone to being overlooked. Through this review presenting two cases seen in our rheumatology clinic, our aim is to raise awareness about calciphylaxis which can mimic vasculitis, and promote early diagnosis.

## INTRODUCTION

Calciphylaxis, which literally means ‘protection through calcification’, is a fatal disease characterised by ischemic necrosis of cutaneous tissue resulting from vascular intimal fibroplasia, thrombi, and calcifications in the arterioles. The disease generally presents with painful skin ulcers and necrosis as its primary manifestations.^1^ Studies have shown that the disease can carry a mortality rate ranging from 33% to 80%.^2^ The pathophysiology involves the accumulation of calcium in the skin, vascular space, and adipose tissue due to abnormal serum levels of calcium, phosphorus, and parathyroid hormone (PTH), particularly in patients with end-stage renal disease (ESRD), a condition known as calcific uremic arteriolopathy (CUA). Additionally, there is a propensity for thrombosis in calcified vascular areas, often exacerbated by accompanying vitamin K deficiency. In addition to CUA, characterised by metastatic calcifications in patients with ESRD and high calcium and phosphorus levels, there are also cases of non-uremic calciphylaxis, although they are less frequent.^3^ Key risk factors for calciphylaxis include vitamin K deficiency, deficiencies in protein C and S, low serum albumin levels, being of white race, female gender, and obesity.^4^ Maintaining a high level of clinical suspicion is crucial in diagnosing calciphylaxis.

The emergence of purple-black lesions in the form of painful, scarring, and ulcerated plaques, particularly in patients with ESRD or undergoing renal replacement therapy (RRT), serves as a significant warning sign for physicians. While abnormal serum calcium, phosphorus, and PTH levels are commonly observed in these patients, the absence of abnormalities in these markers does not rule out the disease. Additionally, some clinics rely on histopathological findings for diagnosis, with punch biopsy being the preferred method for examining skin lesions.^5^ The clinical presentation typically involves severe ischemic and neuropathic pain, which may occur independently of skin lesions. Patients may exhibit a range of skin lesions, including livedo reticularis, plaques, nodules, and ulcerations, with a predilection for trauma-prone areas such as the thighs and abdomen. These lesions pose a significant risk to patients due to the potential development of secondary infections.^2^

In this study, we report two new cases of calciphylaxis encountered in our rheumatology clinical practice. Additionally, we aim to draw attention to calciphylaxis by compiling and analysing cases reported in the literature.

## CASES

**Figure 1.**
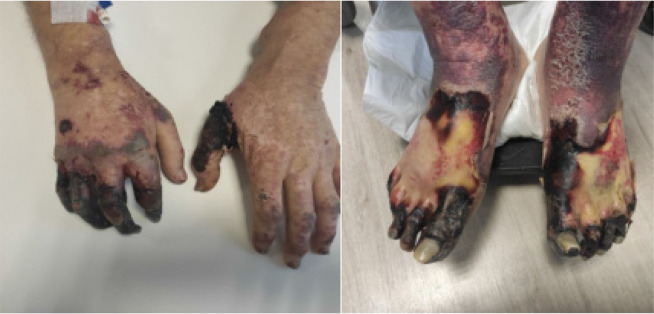
Widespread necrotic lesions around feet and hands of case one.

**Figure 2.**
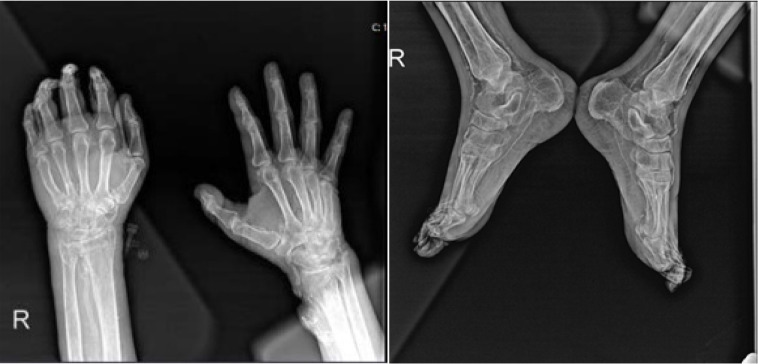
Hand and feet X-rays showing calcifications (shown with arrows).

Our first case involved a 53-year-old male patient with a medical history significant for hypertension, ulcerative colitis, and two prior kidney transplantations, which were rejected ten years and four years ago, respectively. He also had ESRD and was undergoing RRT. The patient presented to our rheumatology outpatient clinic with newly developed pain in both hands and distal feet, along with widespread necrotic wounds resembling vasculitic lesions (**Figure 1**). The serological examination revealed that the antinuclear antibody (ANA) level determined with indirect immunofluorescence assay (IFA) was positive at a 1/160 titre (coarse spotted, mid body), while the extractable nuclear antigen (ENA) profile, antineutrophil cytoplasmic antibodies (ANCA), and anti-phospholipid (APA) antibodies were negative. Radiographs of the patient revealed widespread calcifications around the radial and posterior tibial arteries (**Figure 2**). Rheumatological pathology was not initially considered for the patient. During this period, widespread necrotic wounds developed on his hands and upper extremity doppler USG revealed triphasic flow along with wall calcific plaques in the lumen in the right axillary and brachial arteries (preliminary diagnosis of thrombosis or Buerger’s disease). The patient was initiated on antiplatelet and anticoagulant therapies with preliminary diagnosis of calciphylaxis. Serum electrolyte and PTH levels were requested for the patient. The results were as follows (values with normal limits in parentheses): urea 136 mg/dL (10–40 mg/dl), creatinine 9,8 mg/dL (0.5–1.4 mg/dl), calcium 8.7 mg/dL (8.5–10.5 mg/dl), phosphorus 9 mg/dL (2.8–4.5 mg/dl), white blood cell count (WBC) 16.10^3^/mm^3^, (4.5.10^3^/mm^3^−10.10^3^/mm^3^), platelet count (PLT) 222.10^3^/mm^3^ (150–450.10^3^/mm^3^), and PTH level 1800 pg/mL (15–65 pg/ml). Following these findings, treatment with 30 mg of cinacalcet was initiated. The patient was planned to be started on sodium thiosulfate (STS); however, it was not available in our country. During follow-up, the patient developed hypotension and fever within ten days of diagnosis and necrotic wounds showed progressive expansion. Multiple antibiotics were administered, and the patient was monitored in intensive care with preliminary diagnosis of sepsis. Unfortunately, despite treatment efforts, the patient succumbed to the illness one month after being diagnosed with calciphylaxis.

**Figure 3.**
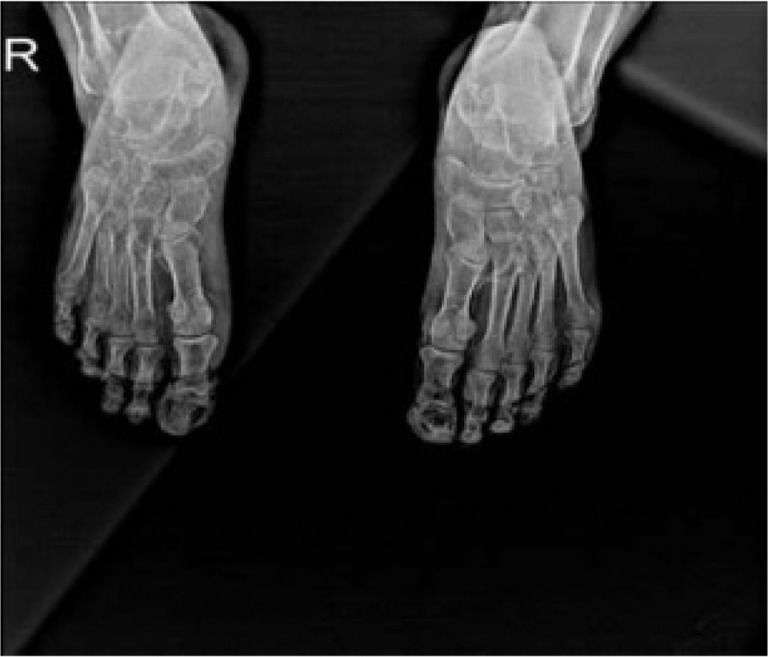
Feet X-ray showing calcifications (showed with arrows) of our second case.

In the second case, a 49-year-old male patient with a medical history of hypertension referred to our rheumatology outpatient clinic with worsening pain, ecchymosis, and oedema in the upper extremities over the past 1.5 months with preliminary diagnosis of vasculitis. The patient had undergone two kidney transplants, the first fourteen years ago and the second seven years ago, following seven years of peritoneal dialysis. Upon examination, a filling defect consistent with thrombus was identified in the proximal bilateral radial artery and distal part of the left ulnar artery during upper extremity Doppler USG. As a result, the patient was consulted with the cardiovascular surgery clinic. Antiplatelet, anticoagulant, and iloprost therapies were initiated. Upon examination, ecchymotic lesions were observed on the hands and feet. The patient’s laboratory results were as follows: urea 164 mg/dL, creatinine 5,07 mg/dL, calcium 9,88 mg/dL, phosphorus 5,16 mg/dL. WBC was 5,34.10^3^/mm^3^, PLT was 140.10^3^/mm^3^. ANA, ENA profile, ANCA and APA antibodies yielded negative results. The radiographs of feet obtained from the patient were assessed as indicative of calciphylaxis (**Figure 3**). Although a biopsy from the distal upper extremity was recommended, the patient declined to undergo the procedure. Following diagnosis, iloprost therapy was continued. Subsequently, a significant regression was observed in the lesions.

## DISCUSSION

Calciphylaxis is characterised by calcification and fibrosis of the media layer of the arterioles, intermittent giant cell formation, intimal proliferation, intraluminal thrombosis, and secondary necrosis. It was first described in 1962.^6^ Its frequency is estimated to be around 1% in patients diagnosed with chronic kidney disease (CKD) and 4% in those with ESRD, with a 1-year mortality rate of 45–80%.^7^ Calciphylaxis in the absence of ESKD is called non-uremic calciphylaxis (NUC). In one systematic review on NUC, which included 36 cases, the mortality rate was 52%, which was similar in cases with CKD. NUC was defined especially in white women, with sepsis being the leading cause of death.^8^ In another review in 2024, a case study revealed a mortality rate of 83% at 12 months, with 92% of these patients suffering from CUA, while a single patient belonged to the NUC cohort.^9^ As far as can be found in the literature on non-uremic calciphylaxis, warfarin-associated calciphylaxis has a more favourable prognosis with a lower mortality rate (17%) compared to uremic calciphylaxis.

While increasing calcium, phosphorus, and PTH levels are believed to be the primary triggers, the progression of calciphylaxis may persist even after these levels return to normal. Given the challenging nature of treatment and the associated high morbidity and mortality rates, the prevention and early detection of calcification are of paramount importance. Regarding skin lesions, APA syndrome, vasculitides, dystrophic calcification are some other diseases that should be considered in patients presenting with painful skin lesions, ulcerations, and thrombosis.

As there are no specific diagnostic criteria or laboratory tests, the disease is primarily recognised based on clinical findings. While elevated serum calcium/phosphorus and PTH levels serve as warning signs, radiological, clinical, and, if necessary, histopathological evaluations should be conducted collectively. However, due to the risk of secondary infection and ulcer spread associated with the current immunosuppression in patients, caution should be exercised when considering biopsy. Calcific panniculitis with septal involvement is commonly observed on biopsy.^10^

Studies have identified diabetes mellitus, obesity, and the use of vitamin K antagonists as risk factors.^11^ Hence, given that elevated calcium/phosphorus and PTH levels contribute to vascular microcalcifications, parathyroidectomy should be considered in suitable patients. Additionally, it’s crucial to avoid indiscriminate calcium and vitamin D supplementation to prevent secondary hyperparathyroidism in CKD patients, manage hyperphosphatemia effectively, and exercise caution when prescribing anticoagulants, particularly vitamin K antagonists, to individuals with risk factors. Lifestyle modifications and dietary changes are also essential interventions. Treatment should be multidisciplinary, involving close monitoring for ulceration and proactive measures to prevent superinfection, necrosis, or spread of the lesions.^12^ To achieve this goal, strategies may include increasing the frequency of weekly sessions to 4–5 in patients undergoing RRT, transitioning patients on peritoneal dialysis to haemodialysis, implementing dietary phosphorus restriction along with the use of oral phosphorus binders, discontinuing vitamin D supplementation, avoiding extremes in PTH levels, reevaluating the use of oral anticoagulants such as warfarin, and considering the use of calcium chelators. Comprehensive wound care and effective pain management are also fundamental components of treatment.^3^ Achieving sufficient pain relief is challenging due to the complexity and severity of pain, often necessitating pain management consultation. The exact mechanisms behind the pain associated with calciphylaxis are not fully elucidated, but it is believed that both ischemic and neuropathic factors may be involved. In patients with calciphylaxis, early surgical debridement is crucial, as it is correlated with greater six-month survival rates in comparison to cases where debridement is not undertaken. The local management of wounds should consist of clearing wound debris, applying non-adhesive dressings, and using antiseptic or antimicrobial agents as required.^13^

In terms of wound surgery, the use of aggressive surgical wound debridement remains a subject of debate in calciphylaxis treatment. Some surgical interventions for investigative or therapeutic purposes, including incisional biopsy, drainage procedures, and even insulin injections, have been reported to potentially trigger calciphylaxis. Considering that sepsis caused by infected wounds is the leading cause of mortality, it is imperative to employ aggressive wound care to prevent infection of necrotic tissue or eschar. Surgical or chemical debridement for removing necrotic tissue has been advocated in various studies.^14^

STS can be utilised to neutralise reactive oxygen radicals and decalcify the vascular bed. STS has been demonstrated to be effective for treating calciphylaxis in dialysis patients. Additionally, meta-analysis studies have shown its effectiveness in reducing coronary artery calcification, a common morbidity in ESRD, among dialysis patients. STS prevents the formation and precipitation of calcium deposits by converting calcium into calcium thiosulfate, a highly soluble compound. Despite numerous reports discussing treatment mechanisms including calcium-chelating properties, antioxidative effects, and STS-induced changes in serum inhibitors of vascular calcification, the exact mechanism of STS remains unclear, and the concentrations needed for these effects are yet to be determined.^15^

While there may not be sufficient studies available, some research has demonstrated the benefits of cinacalcet use. Cinacalcet, a class II calcimimetic that targets the calcium-sensing receptor of the parathyroid gland chief cells, can be used to treat the patient’s secondary hyperparathyroidism. Cinacalcet alters the calcium receptor and increases its sensitivity to calcium. It is prescribed for the treatment of both secondary hyperparathyroidism in patients undergoing haemodialysis and hypercalcemia in patients with parathyroid carcinoma.^11^

Hyperbaric oxygen therapy (HBOT) has shown promise in aiding the healing of necrotic wounds associated with calciphylaxis by preventing phagocytosis. The anticipated advantages span across diverse clinical scenarios united by a shared origin of tissue hypoxia and necrosis. HBOT facilitates oxygen delivery to hypoxic tissues and fosters wound healing by stimulating fibroblast proliferation and angiogenesis. By preventing phagocytosis, it also restricts the spread and advancement of infection by enhancing oxygen-dependent neutrophil bactericidal activity.^16^

Bisphosphonates are also emerging as an alternative approach for patients with CUA. The mechanisms responsible for the therapeutic effects of bisphosphonates on CUA remain unknown, although several possibilities have been proposed. Pharmacologically, bisphosphonates alter crystal growth or calcium hydroxyapatite reabsorption in vitro, depending on their concentration. It is hypothesised that a similar effect occurs in vivo.^17^

The primary aim of using corticosteroids in calciphylaxis is to reduce tissue inflammation, although the treatment approach remains controversial. On the one hand, corticosteroids may be linked to the development of calciphylaxis and an elevated risk of infection in ulcerated lesions. On the other hand, this medication reduces tissue inflammation and has demonstrated effectiveness in certain cases. Absolutely, caution is warranted when contemplating the use of corticosteroids for treating calciphylaxis.^18^

A review of the English literature on PubMed with the terms ‘calciphylaxis’, ‘uremic calcific arteriolopathy’, ‘vasculitis’ yielded 34 cases of calciphylaxis. With the addition of the two cases reported in this study, the total number of cases amounts to 36 (**Table 1**). Among these cases, 16 are women. Previous studies have reported a higher frequency of calciphylaxis in women compared to men. This compilation represents the first comprehensive study on calciphylaxis in Türkiye. Upon examination of the cases, a mortality rate of 33% is observed despite appropriate treatment and care. Furthermore, 91% of deaths are associated with sepsis. Hence, wound care and infection management play a vital role in the treatment of calciphylaxis. Given its rarity, calciphylaxis is prone to being overlooked. However, this review highlights that mortality and morbidity rates can be significantly reduced when calciphylaxis cases are detected and treated before necrotic wounds develop. Therefore, early diagnosis is crucial. Through this review, our aim is to raise awareness about calciphylaxis which can mimic vasculitis in clinical practice and promote early diagnosis.

**Table 1.** Literature cases up to date.^19–39,40,41^

	**Non-uremic calciphylaxis (NUC) n=12**	**Uremic calciphylaxis (CUA) n=24**
**Epidemiological characteristics**		
Female sex, n(%)	10 (80)	6 (25)
Mean age	60,75	43,5
**Comorbidities, n(%)**		
Diabetes mellitus	4 (33)	4 (16)
Hypertension	4 (33)	7 (29)
Heart failure	2 (16)	2 (8)
Obesity	5 (41)	2 (8)
Hyperparathyroidism	5 (41)	2 (8)
Atrial fibrillation	5 (41)	4 (16)
Multiple sclerosis	0 (0)	1 (4)
Heart valve replacement	1 (8)	1 (4)
Renal trans history	0 (0)	4 (16)
Deep vein thrombosis	1 (8)	0 (0)
Polymyalgia rheumatica	1 (8)	0 (0)
Rheumatoid arthritis	1 (8)	0 (0)
**Treatment modalities, n(%)**		
Sodium thiosulfate	7 (58)	11 (45)
Corticosteroids	8 (66)	3 (12)
Hemodialysis	1 (8)	8 (33)
Lenalidomid	1 (8)	0 (0)
Skin graft, surgery, amputation	4 (33)	3 (12)
Hyperbaric oxygen therapy	2 (16)	1 (4)
Phosphorus binding agents	1 (8)	3 (12)
Rivaroxaban, dabigatran	2 (16)	0 (0)
Pentoksifylline, iloprost	1 (8)	1 (4)
Heparin, enoxaparin	5 (41)	5 (20)
Warfarin	7 (58)	6 (25)
Vitamin D	2 (16)	2 (8)
Cinacalcet	3 (25)	3 (12)
Bisphosphonates	5 (41)	2 (8)
**Survival n(%)**		
Remission	8 (66)	17 (70,8)
Ex	4 (33)	7 (29)

## CONCLUSION

While preventing the development of calciphylaxis as a vasculitis mimic in patients with risk factors is crucial, treatment strategies are also employed to manage existing cases. However, treatment decisions are challenging due to the rarity of calciphylaxis, the lack of a specific treatment protocol, and the variability in approaches used in current cases. It is imperative to conduct more clinical studies in the future to establish a standardised treatment protocol and identify new modalities for both treatment and prevention.

## FUNDING

No funding.

## CONFLICT OF INTEREST

The authors declare no conflicts of interest.
